# A Melodic Contour Repeatedly Experienced by Human Near-Term Fetuses Elicits a Profound Cardiac Reaction One Month after Birth

**DOI:** 10.1371/journal.pone.0017304

**Published:** 2011-02-23

**Authors:** Carolyn Granier-Deferre, Sophie Bassereau, Aurélie Ribeiro, Anne-Yvonne Jacquet, Anthony J. DeCasper

**Affiliations:** 1 Laboratoire de Psychologie et Neuropsychologie Cognitives, CNRS-FRE 3292, Université Paris Descartes, Paris, France; 2 Laboratoire Psychologie de la Perception, CNRS-UMR 8158, Université Paris Descartes, Paris, France; 3 Department of Psychology, University of North Carolina at Greensboro, Greensboro, North Carolina, United States of America; Université Pierre et Marie Curie, France

## Abstract

**Background:**

Human hearing develops progressively during the last trimester of gestation. Near-term fetuses can discriminate acoustic features, such as frequencies and spectra, and process complex auditory streams. Fetal and neonatal studies show that they can remember frequently recurring sounds. However, existing data can only show retention intervals up to several days after birth.

**Methodology/Principal Findings:**

Here we show that auditory memories can last at least six weeks. Experimental fetuses were given precisely controlled exposure to a descending piano melody twice daily during the 35^th^, 36^th^, and 37^th^ weeks of gestation. Six weeks later we assessed the cardiac responses of 25 exposed infants and 25 naive control infants, while in quiet sleep, to the descending melody and to an ascending control piano melody. The melodies had precisely inverse contours, but similar spectra, identical duration, tempo and rhythm, thus, almost identical amplitude envelopes. All infants displayed a significant heart rate change. In exposed infants, the descending melody evoked a cardiac deceleration that was twice larger than the decelerations elicited by the ascending melody and by both melodies in control infants.

**Conclusions/Significance:**

Thus, 3-weeks of prenatal exposure to a specific melodic contour affects infants ‘auditory processing’ or perception, i.e., impacts the autonomic nervous system at least six weeks later, when infants are 1-month old. Our results extend the retention interval over which a prenatally acquired memory of a specific sound stream can be observed from 3–4 days to six weeks. The long-term memory for the descending melody is interpreted in terms of enduring neurophysiological tuning and its significance for the developmental psychobiology of attention and perception, including early speech perception, is discussed.

## Introduction

Human hearing develops progressively during the last trimester of gestation. By 35weeks Gestational Age (GA), cochlear biomechanics and frequency selectivity are mature and absolute auditory thresholds are about 10 dB Hearing Level in the premature infant [e.g., 1]–[4]. Near-term characteristics of fetal cardiac responses to airborne sounds demonstrate that fetuses can discriminate intensity, frequency and spectra [5]–[11] and can also process some fast and slow amplitude temporal variations in auditory streams [12], [13], [14]; see [13], [15] for extended reviews. Fetal MEG studies, using the Mismatch Negativity paradigm with tone bursts, confirmed that detection of frequency changes occurs *in utero* [e.g., 16]–[19] and shows that it happens very early in development, at 28 weeks GA [17], [19], therefore only 2–3 weeks after the onset of cochlear function [1]. Auditorially evoked cortical activation has been confirmed with fMRI in the near-term fetus [20]–[21], and as early as 33 weeks GA [22]. Learning studies (see below) indicate that fetuses can also perceive temporal variations in the spectra and in amplitude of complex auditory streams such as speech sequences.

For airborne sounds recorded within the amniotic fluid in the gestating ewe [e.g., [23] –[24]] and in women during delivery [25]–[28], power-spectrum analyses show that most components over 60 dB SPL (Sound Pressure level, re: 20 µPa) in the mother's near field environment are transmitted with little distortion into the uterus and, in general, are not masked by internal sounds. Frequencies ≤0.4 kHz are not attenuated; attenuation increases with frequency at a rate of about 6 dB/octave but never exceeds 30 dB above 4 kHz. The transfer functions of complex sounds are themselves more complex and their overall attenuation is lower than that of tones and band noises, e.g., voice attenuation depends on external SPL [29], music is attenuated ≤10 dB SPL in the gestating ewe [30]. In contrast, the maternal voice itself suffers no or little attenuation in the womb [25]–[28].

Near term fetuses and newborns can remember simple and complex sounds that frequently occurred earlier in prenatal life. Here, we will refer to fetal auditory memory when fetuses or infants respond to a sound they had previously and frequently experienced before birth differently than do non-exposed fetuses or infants. It has been examined in three developing psycho-biological domains [31], state or fetal motility, cardiac autonomic responses and associative learning. The state modifications investigated in the fetus were change from a non-active to an active state, habituation rate of gross body movements, changes in behavioral state and state transitions. In the neonate, state changes include the passage into a quiet-alert state, orientation and attention to the stimulus. The cardiac response examined was cardiac deceleration. The associative learning that was studied includes classical conditioning of movements in the fetus, and operant learning in the newborn. These responses differ in their degree of reflexivity, sensory-motor integration, precision of the motor behavior engaged and auditory processing required to resolve the stimulus (stimulus complexity). Memories have been found up to three-four days after birth and prenatal memory effects beyond this period is unknown. Our study assessed memory for a melodic sequence that fetuses repeatedly experienced *in utero* after a 6-week retention interval, when they were 1-month old infants. A review of the data supporting the phenomena of prenatal auditory memory in three psycho-biological domains follows.

### Prenatal studies

Habituation of fetal gross body movements and heart rate responses to loud airborne noises and tones [e.g., 32]–[34], or vibro-acoustic stimuli placed against the mother's abdomen [e.g., 35]–[41] have been extensively studied mostly for clinical reasons since the early eighties. Prenatal memory has been mostly investigated by comparing habituation and re-habituation rates of gross movements. Vibro-acoustic studies show a savings in the number of trials to re-habituation after a 10 min delay in fetuses ≥30 weeks GA, independently of GA, after a 24 hours delay [e.g., 42]–[43], a 4-week interval with first habituation at 34 weeks GA [43], or 1–2 days after birth when habituation occurred in the days before birth [44]. It should be noted here that vibro-acoustic stimuli provide tactile and auditory stimulations to the mother and auditory, tactile, proprioceptive and vestibular stimulations to the fetus. Most stimulators have a wide frequency band with fundamental frequencies and first overtones ranging from 75 to 300 Hz. They have a medium-high SPL in air (70–80 dB) but they are highly amplified inside the amniotic fluid, e.g., ≥110 dB intra-uterine level in the gestating ewe [45]–[46]. Abrams et al. found that the ranges of the fetal lamb head accelerations were proportional to the vibrators' SPL inside the amniotic fluid [46]. Therefore, they do not give any clear information about prenatal auditory learning per se. Perhaps, a real-world expression of long term habituation to loud noises is provided in the studies done near Osaka's International Airport: Infants of mothers who lived there before the third trimester of pregnancy did not wake up and had little or no EEG reaction during sleep to a recorded aircraft noise at 80 dB SPL but were awakened by an 80 dB SPL music sequence that had similar spectra [47]. Two experiments indicating classically conditioned movements, with startling noise or a vibro-acoustic stimulus as the unconditioned stimulus and milder vibro-acoustic or tone stimuli as the conditioned stimulus, were reported during the last 2-months of gestation [34], [48]. They suggest that prenatal memory can last several weeks. For example, successful conditioning was reported in 32–39 weeks GA fetuses and successes were independent of GA [34]. Interestingly, such association was shown in a fetal chimpanzee and the conditioned response was observed two months after birth [49].

Fewer studies have examined the effect of repeated exposure to complex auditory streams within the fetal period. Musical stimuli are reported to elicit an increase in movements [34] or in mean Heart Rate (HR) and HR variability in familiarized fetuses compared to control fetuses [50]. Two studies investigated prenatal auditory memories when mothers recited a specific speech passage aloud each day, one between the 35^th^–38^th^ weeks GA [51] and another from 28–34 weeks GA [52]. When tested with a tape recording of their target stories and a control story, the target stories elicited a brief cardiac deceleration and the control story did not. The stories were emitted at a low SPL that does not usually evoke a HR change. In both cases, differential responding elicited by the target passage was independent of the speakers' voice used during the test. Other studies showed that the heart rate of fetuses exposed to a long recording of their mother's voice or a female stranger's voice saying the same speech passage are different [e.g., 53]–[54].

### Postnatal studies

Most prenatal auditory learning studies in the newborn used various versions of the maternal voice, speech, language and musical stimuli as reinforcers in operant learning tasks. Operant choice procedures with newborns can tell us whether and which characteristics of a sound can affect its power to reinforce behavior (for the cognitive implications of newborn operant learning, see [55]). They are most informative because they directly oppose the reinforcing power of prenatal sounds against that of control sounds. Two different procedures have been used that allow the newborn to express a choice between two sound stimulations, the differential reinforcement procedure and the stimulus discrimination procedure. In the differential reinforcement procedure, a short-latency sucking response will cause a sound (A) to occur and a long-latency sucking response will cause another sound (B) to occur. The infant can make either response at any time, but after learning the infant will choose to make the response that causes the more reinforcing sound to occur more often. In the stimulus discrimination procedure, two arbitrary brief sounds (e.g., tones, vowels), S1 and S2, occur in a random sequence, e.g., S1, S2, S2, S1. S1, S1, S2…. If the infant makes a sucking response when S1 is present, the response causes S1 to go off and a sound (A) to occur. If the infant makes the response when S2 is present the response causes S2 to go off and another sound (B) to occur. S1 and S2 occur randomly and independently of the infant's behavior and the infant can choose to suck at any time. After learning, the infant chooses to suck more often during the brief sound that signals the sucking response will cause the more reinforcing sound to occur more often. With the differential reinforcement procedure the infant learns which of two precise motor responses will cause sound A or B to occur. With the stimulus discrimination procedure the infant learns when to make a response that will cause sound A or B to occur. The difference in reinforcing power is expressed by the newborn's preference to trigger one reinforcer over the other.

Eleven operant choice studies have been conducted with newborns less than 72 hours after birth. In summary they found that newborns chose or prefer: their mother's voice vs. stranger's female voice [56], a low pass filtered version of the mother's voice, vs. the airborne version [57]–[59], a specific speech passage [60] and melody [61] their mother frequently recited or sang only while pregnant over novel speech passage/melody. They also prefer their mother's language over a different language they never heard before [62]–[64] and sentences from both languages of bilingual women were found to be more reinforcing for their infants than a non-maternal language [64]. Thus, operant learning procedures show that previous exposure to specific maternal vocalizations influenced their capacity to differentially reinforce learning of specific fine motor responses. Significantly, as in the fetus, newborns' memory of the maternal voice is independent of what she is saying, and their memory of the maternal language, a speech passage frequently repeated or a melody often sang, is independent of the voice of the speaker. The effect of emotional speech patterns of the maternal language was investigated with a non operant procedure. Newborns have more eye openings during happy speech in the maternal language than during regular speech, but not with a foreign language [65]; this suggests memory of prosodic features of the maternal language. Some authors have argued that early postnatal experience rather than prenatal experience could explain some of these results. However, where the prenatal sounds were experimentally manipulated, there is little concern that postnatal experience might have affected the newborn's behavior.

Other works showed that a musical stimulus experienced before birth can alter behavioral states [50] or trigger specific attentional response in newborns. In a naturalistic study, Hepper [66] showed that newborns went quickly into a quiet-alert attentive state upon hearing the jingle from a TV soap opera that their mothers' watched daily throughout their pregnancy, but not upon hearing the backward version of the jingle or control music.

This review indicates that prenatal memory is present very early, from about 30 weeks GA, and that near-term fetuses register some of the spectral and temporal features of recurrent complex stimuli, e.g., prosody, melodic contour of speech, language and music. These memories can affect very different psychobiological domains, from gross body movements, to orienting responses, to operant discrimination learning in newborns, and that their impact is carried into the newborn period. However, prenatal memory effects beyond this period have not been observed. Hepper [34] attempted to replicate the state-altering effects of the TV jingle (cited above) when the infants were 21 days old and had insured that the mothers did not watch the TV program after delivery. The attempt was unsuccessful. He noted that either long-term prenatal memory does not exist or that the prenatal experience was insufficiently controlled and/or that the dependent variable was insensitive to the effects of the prior experience.

In this study, we assessed prenatal auditory memory for a descending contour melody after a 6-week retention interval. Fetuses were exposed to the melody twice daily between their 35^th^ and 38^th^ week of gestation under precisely controlled conditions. When 1-month old (44 weeks GA), they and a control group were stimulated one time, in quiet sleep, with the descending melody or with an ascending control melody. Both melodies were composed specifically by Claire Gérard (Université de Poitiers, France) so as to differ mainly in the direction of their contour; they had very similar spectra, almost identical amplitude envelopes but precisely inverse melodic contours. The two melodies had been previously shown to reliably elicit similar HR decelerations in 37–38 weeks GA fetuses during quiet sleep [13]. We selected melodic contour as the dimension of interest because it is an important characteristic of speech and probably the most salient feature of music for infants [e.g., 67]. We selected a descending contour for the Experimental melody because it is less attractive to infants than an ascending contour, a major feature of “motherese” [e.g., 68]. We assessed the effect of prenatal exposure to the descending melody by examining the direction and magnitude of the cardiac response elicited by the two melodies in both exposed and naive infants. Our results extended the retention interval over which a prenatally acquired memory of a specific sound stream can be observed from 3–4 days to six weeks.

## Results

### Number and direction of the cardiac responses

All 50 infants tested showed a stimulus elicited HR change. The stimuli evoked small startle movements accompanied by HR acceleration in 18 of them: 10 infants (40%) in the Control Group and 8 infants (32%) in the Experimental Group. The number of accelerations did not differ between groups, χ^2^ (1) = 0.35, p = 0.56. Because our interest was in the cardiac orienting or cardiac attention response, not in startles, i.e., somato-cardiac coupling effects, HR accelerations were not analyzed any further. All other infants (n = 32) showed a cardiac deceleration to the melodies. In the Control Group, 60% of the infants (nine boys and six girls) showed a cardiac deceleration; six had the Control Melody and nine the Experimental Melody. In the Experimental Group, 68% of the infants (9 boys and 8 girls) showed a cardiac deceleration; nine had the Control Melody and eight the Experimental Melody. There was no significant difference in the number of cardiac decelerations between groups as a function of the melodies, χ^2^ (1) = 0.54, p = 0.46. Deceleration responses were examined in detail.

### Heart Rate analysis of the subjects with a cardiac deceleration

HR during the 15 s Prestimulus Period and the 15 s Stimulation Period were analyzed separately with a mixed ANOVA, having Group (Experimental vs. Control) and Melody (Experimental vs. Control) as between factors and Time (1–150) as the within factor.

#### Prestimulus Period

**Figure 1:**
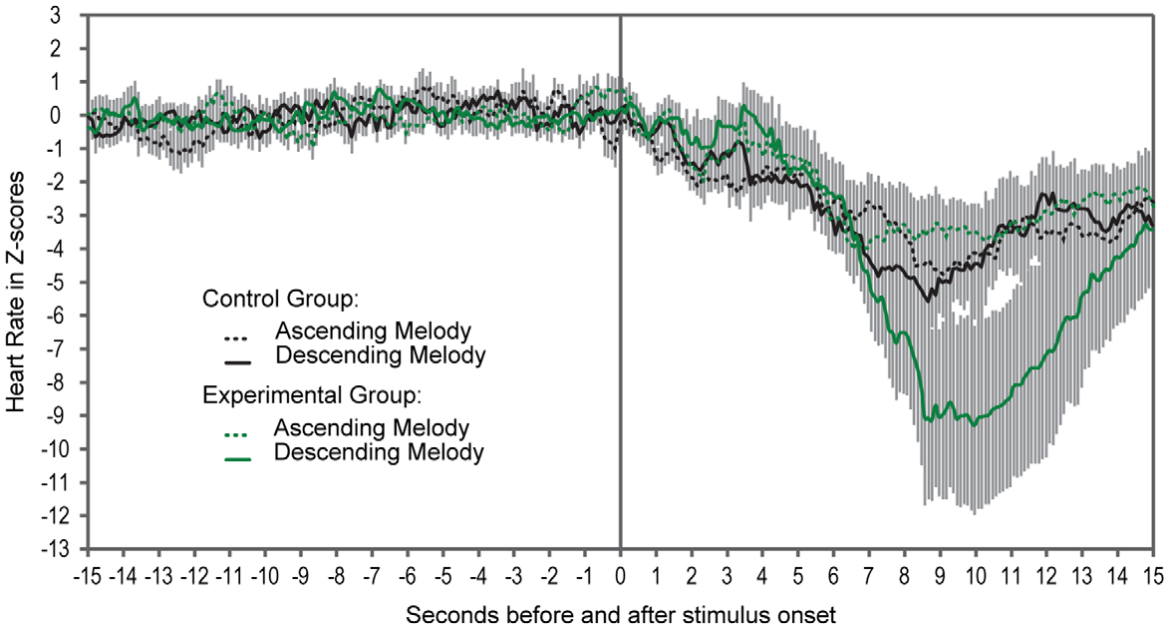
Mean Heart Rate ± sem, in Z scores, for the 300 Heart Rate values (10/s) during the last 15 s of the Prestimulus Period and the following 15 s Stimulation Period.

The mixed ANOVA was conducted on HR in bpm. The main effects of Group, F (1, 28) = 1.57, p = .22; Melody, F (1, 28) = 2.08, p = .16 and the Group x Melody interaction, F (1, 28) = 0.24, p = .63, were not significant. The main effect of Time was significant, F (149, 4172) = 1.26, p = .018, but all interactions involving Time were not: In fact, all F-values were <1.0 and had p-values between .59 and .91. This analysis indicates that HR did not differ between the 4 SubGroups during the 15 s Prestimulus Period, but small unsystematic moment to moment variations occurred in all groups (see Table 1 and Figure 1).

**Table 1:** Mean Heart Rate (HR) ± SD in beats/min and Mean Heart Rate Variability (HRV) (i.e., Mean Standard Deviation) ± SD during the 15 s Prestimulus Period for each of the 4 SubGroups.

Group	Melody	HR ± SD	HRV ± SD
Cont. Gr.	Exp. M.	146.53±8.19	1.30±0.40
	Cont. M.	140.51±9.6	1.26±0.64
Exp. Gr.	Exp. M.	141.11±10.90	1.35±0.39
	Cont. M.	138.14±5.93	1.40±0.48

Cont.Gr. (Control Group); Exp.Gr. (Experimental Group);

Exp.M. (Experimental Melody: Descending);

Cont.M. (Control Melody: Ascending).

#### Stimulation Period

For each subject, the 150 (10/s) digital HR values during the 15 s Stimulation Period were converted to z-scores based on the subject's mean and standard deviation during the 15 s Prestimulus Period. The normalization controls for individual differences in baseline mean and variability that can affect the amplitude of the stimulus-elicited HR changes [8], [69]. The mixed ANOVA on the z-scores showed no statistically significant effects of Group, F (1, 28) = .30, p = .59, Melody, F (1, 28) = 1.35, p = .26, or Group x Melody interaction, F (1, 28) = 1.22, p = .28. All effects involving the Time factor were statistically significant: Time, F (149, 4172) = 13.02, p<.00001, η^2^_p_ = .32; Time x Group, F (149, 4172) = 1.38, p<.0017, η^2^_p_ = .05 and Time x Melody, F (149, 4172) = 1.79, p<.00001, η^2^_p_ = .06. Time alone explained a high percentage of variance (32%) because the stimulation always elicited a cardiac deceleration. HRs reached their lowest values between 9 and 10 seconds after stimulus onset (Figure 1). Interpreting the Time x Group and the Time x Melody interactions is impossible because some members of both the Experimental and Control Groups were stimulated with the Control Melody and the Experimental Melody. However, and most importantly, the Time x Group x Melody interaction was also statistically significant, F (149, 4172) = 1.56, p<.00003, η^2^p = .053. Figure 1 indicates this interaction occurred because HR decelerations elicited from the Experimental infants by the Experimental Melody reached an average maximum of z = −9.28, which is 3–5 SD lower than the lowest average maximum HR level reached by the Experimental infants tested with the Control Melody (z = −4.00), the Control infants tested with the Experimental Melody (z = −5.57) and the Control infants tested with the Control Melody (z = −4.71).

This interpretation is supported by three separate mixed ANOVAs with SubGroup (Experimental Group tested with the Experimental Melody vs. another SubGroup) and Time (1 – 150) as factors. In each case the main effect of SubGroup was not significant, the F-values for df (1, 15) and df (1, 12) ranged between 0.81–1.94 and their p-values ranged between .18–.39. In each case the Time factor was statistically significant, because the stimuli always elicited a cardiac deceleration. Most importantly, all SubGroup x Time interactions were also statistically significant. When the Experimental SubGroup tested with the Experimental Melody was contrasted with the Experimental SubGroup tested with the Control Melody the F- statistics were: Time, F (149, 2235) = 8.76, p.<.00001, η^2^_p_ = .37, and SubGroup x Time interaction, F (149, 2235) = 2.48, p.<.00001, η^2^_p_ = .14. For the contrast with the Control SubGroup tested with the Experimental Melody, the F- statistics were: Time, F (149, 2235) = 8.37, p.<.00001, η^2^_p_ = .36 and SubGroup x Time interaction, F (149, 2235) = 1.92, p.<.00001, η^2^_p_ = .11. For the contrast with the Control SubGroup tested with the Control Melody the F- statistics were: Time, F (149, 1788) = 6.22, p.<.0001, η^2^_p_ = .34 and SubGroup x Time interaction, F (149, 1788) = 1.73, p.<.0001, η^2^_p_ = .13. When these three SubGroups were assessed together in a mixed ANOVA, only the main effect of Time was statistically significant, F (149, 3129) = 9.40, p.<.0001, η^2^_p_ = .31. The F values for the effect of SubGroup, F (2, 21) = .24, and the SubGroup x Time interaction, F (298, 3129) = .44 had P values ≥.79. The results of the non-parametric Mann-Whitney U test were consistent with those of the parametric analyses. The maximum HR deceleration value reached by Experimental infants tested with the Experimental Melody was significantly lower than those of the infants in the three other SubGroups pooled together, U = 52, p = .028, 1-tail test.

In sum, statistical analyses indicated that there were no differences between the HRs of the four SubGroups during the Prestimulus Period and that onset of the melodies elicited a significant cardiac deceleration in all SubGroups. The deceleration evoked by the Experimental Melody in Experimental infants was 3–5 SD more profound than the decelerations elicited by the Control Melody in the Experimental infants and by both melodies in the Control infants, which did not differ. We concluded that exposing fetuses to a Descending Melody twice each day during the 35^th^, 36^th^ and 37^th^ weeks of gestation affected their cardiac reactions to that melody after a six-week retention interval, when they were one-month old infants.

## Discussion

The introductory review showed that memory for sounds repeatedly experienced before birth has been observed up to three-four days after birth. Our results demonstrated that the retention interval of a memory established between the 35^th^ and 38^th^ week GA can last at least six weeks. Fetuses were given precisely controlled exposure to a descending piano melody twice daily during the 35^th^, 36^th^, and 37^th^ weeks of gestation. Six weeks later we assessed the cardiac responses of 25 exposed infants and 25 naive control infants, while in quiet sleep, to the descending melody and to an ascending control piano melody. The melodies had precisely inverse contours, but similar spectra, identical duration, tempo and rhythm, thus, almost identical amplitude envelopes. When tested at 1-month of age, 60% of the Control infants and 68% of the Experimental infants displayed a short latency HR deceleration with the two melodies. It peaked below pre-stimulus levels 9–10 s after melody onset and then returned towards baseline, a pattern typical of an orienting response [e.g., 70]. Interestingly, the two melodies elicited the same cardiac response pattern in 38 weeks old naïve fetuses [13]. In the fetus, the mean HR decelerations had a lower magnitude and duration (they peaked at 2–3 SD below prestimulus level 6–7 s after onset, and returned to baseline level within 5 s) than in the infants.

Significantly, the HR of the Experimental Group tested with the Experimental Descending Melody decelerated to a level that was twice as low as the HR decelerations of the Experimental Group tested with the Control Ascending Melody and the Control Groups tested with either melody. The present research contributes to the understanding of early auditory development and learning by demonstrating that a mean of 947 presentations of a specific melodic contour, that ended two weeks before birth, is sufficient to impact the autonomic nervous system six weeks later when the infants were one-month old and in quiet sleep. More precisely, the Experimental fetuses showing a HR deceleration to the Experimental Descending Melody had a mean of 39.47, SD = 1.97 (range = 36 – 42) familiarization sessions. Thus, the mean number of presentations of the Experimental Melody was 947 (range = 864 – 1008), among them 631 presentations at 80 dB SPL. The mean retention interval between the end of the exposure period and postnatal testing was 43.47 days, SD = 1.37 (range = 41–46 days).

This result and the research cited in the introduction indicate that recurring auditory experience during human prenatal development of the auditory system can impact multiple psychobiological systems. It produces quantitative, not qualitative, differences in the behavior, e.g., magnitude of reinforcer effectiveness in newborns or the magnitude of HR decelerations seen here. These quantitative differences in behavior suggest that a major or common effect of recurring prenatal experience with a sound is an enhanced subsequent perceptual sensitivity to the sound. In this study, it was not the spectra of musical notes, or the tempo, rhythm, duration of the melody or its amplitude envelope that was most salient, but rather, the arrangement of those aspects over time, the descending melodic contour. The high amplitude response to the contour implies that it left some kind of memory trace.

One possibility is that the profound cardiac deceleration represents a correlate, mediated by the autonomic nervous system, of tuning of the auditory system. The increased sensitivity to various characteristics of a complex sound repeatedly experienced before birth, and observed in different psychobiological domains (see introduction), could be explained by the same underlying neuro-physiological adaptative mechanism. Since the seventies, research with various species, birds and mammals, has revealed specific perceptual and behavioral consequences of particular hearing experiences during the early development of the auditory system [e.g., 71]–[76]. Numerous physiological studies have shown that experience can modify the neuro-functional organization of the developing auditory system, and the coding properties of different auditory structures. The relationships between repeated auditory exposure to a sound and neurocellular modifications of the auditory system is an active area of developmental research; cellular and synaptic plasticity can occur with enhanced receptive field selectivity and enhanced representations in the brainstem and primary auditory cortex for specific features of the stimulus [see 77]–[78 for extended reviews]. Studies showing the effects of repeated exposure to specific sounds and/or temporal variations of simple and complex sounds are clearly relevant to the prenatal human learning studies. For example, in the rat, cortical representation of a tone near threshold can be expanded by repeated mere exposure to the tone [79]; exposure to music enhances auditory detection, sound duration discrimination and alters NR2B protein expression in the auditory cortex [80], and frequency-modulated sweeps increases the number of cortical neurons selective for the rate and frequency direction of the sweep [81].

Perceptual tuning of the auditory system can function as a “physiological memory, [which] is an enduring neuronal change sufficiently specific to represent learned information” [82, p.226]. In humans, tuning could also explain the long-term effect of early auditory experiences, and how, in our study, stimulus specificity persisted in the face of subsequent advances in experience-dependent perceptual development. Our proposal is also consistent with the data described in the Introduction indicating or suggesting that exposure effects can occur very early and can be independent of fetal age after 30 weeks GA [34],[43],[52].

Because there is no, or little attenuation of the mother's voice, speech and language inside the amniotic fluid (see Introduction), many of their regular, non-random, spectral and temporal characteristics are present. It can be estimated that during the same three weeks of exposure to our descending melody, fetuses were also exposed to about 4×10^5^ words spoken by their mother, most of them occurring in normal conversational utterances averaging about 10 words in length [83]–[84]. Maternal vocalizations alone expose the fetal auditory system to an array of speech variations in spectra and amplitude similar to those the newborn will later encounter out of the womb. Interestingly, a simple neural network can acquire the capacity to distinguish English stop consonants spoken by anyone, after mere exposure to exemplars from one speaker that were altered to have the characteristics of speech *in utero* [85]. Furthermore, statistical learning has been demonstrated in the newborn [86]. One can infer that prenatal exposure to maternal speech increases the perceptual salience of the features of an individual mother's voice, speech and language and also to those features that characterize human speech, per se. The idea that auditory tuning during the fetal period may contribute to the development of auditory processing of speech and language and explain the newborn's remarkable discrimination capacities is not new [e.g., 78 ], [87], [88]. Temporal variations, such as the rhythmic properties of speech and language, are considered to play a major role in the development of speech and language perception during infancy [89]. Our results provide the first direct evidence that melodic contours can be processed before birth and remembered for at least several weeks after birth. They are consistent with recent data showing that the contour characteristics of newborns' crying paralleled the main intonation patterns of their maternal language [90].

The potential capacity of physiological tuning to explain a variety of early auditory perceptual phenomena has an almost exact analog in the domain of prenatal chemosensory functional development, perception and memory in animals [e.g., 91]–[92] and humans [e.g., 93]–[94]. Individual infant's specific odor preferences and the often noted human newborn's “bias” toward the odor of human breast milk can “be based on odor information each infant has acquired while in [their own] amniotic fluid” [94, p. 167].

More generally, the memory traces of the fetus' auditory environment in which early maturation of the auditory system occurs can result in the infants' differential responding to specific stimuli. Infants' prenatal auditory experiences can result in either a lack of responsiveness to a postnatal auditory stimulus or, on the contrary, an enhancement of orienting and attention to the stimulus. Physiological memories, i.e., sensory tuning, can bridge the temporal and conceptual gaps in our psycho-biological understanding of the early development of attention, perception, discrimination and recognition of complex sounds before and after birth.

## Materials and Methods

### Subjects

One hundred and twenty five volunteer pregnant mothers with healthy uncomplicated singleton pregnancies participated in the study. They were recruited during prenatal information sessions at Port-Royal Maternity – CHU Cochin-Saint Vincent de Paul University Hospital, Paris. They were briefed about the research and gave informed written consent to participate.

#### Experimental Group

The Experimental Group consisted of 76 infants whose mothers volunteered to listen to a recorded melody each day for three weeks, from the beginning of 35 weeks GA to the beginning of 38 weeks GA, according to instructions. Eighteen infants were not tested because their mothers could not completely follow the prenatal familiarization instructions, or were unable to come to the laboratory at the appointed time. Seventeen infants did not complete testing because they had not entered quiet sleep [95] within the two-three hours allotted (n = 16) or their HR pattern was sinusoidal during the Prestimulus Period (n = 1). Data from sixteen others were rejected after testing because they had many fewer than 35 familiarization sessions (n = 11), their HR signal was lost for more than 2 s (n = 2) or experimenter error occurred (n = 3). The final Experimental Group consisted of 25 infants (13 boys and 12 girls): Twelve of them were tested with the Control Melody (6 girls and 6 boys) and 13 with the Experimental Melody (6 girls and 7 boys).

#### Control Group

The Control Group consisted of 49 infants with no prenatal exposure to the melody. Nineteen infants did not complete testing because they had not entered quiet sleep within two-three hours (n = 15) or their HR pattern was sinusoidal during the Prestimulus Period (n = 4). Data from five others were rejected because the HR signal was lost for more than 2 s (n = 2) or experimenter error occurred (n = 3). The final Control Group consisted of 25 infants (15 boys and 10 girls): Ten were tested with the Control Melody (3 girls and 7 boys) and 15 with the Experimental Melody (7 girls and 8 boys).

All infants in the final samples were born at term, 39–41 weeks GA, with weights appropriate for gestational age and healthy. At the time of postnatal testing, the Experimental and Control Groups did not differ in GA, respectively, Mean = 44 weeks, SD = 2.2 days vs. Mean = 44 weeks, SD = 1.76 days, t (48) = 0.64, p = .52, or in Postnatal Age, respectively, Mean = 30.44 days, SD = 6.36 vs. Mean = 31.48 days, SD = 6.74), t (48) = 0.56, p = .58.

### Sound Stimuli

**Figure 2:**
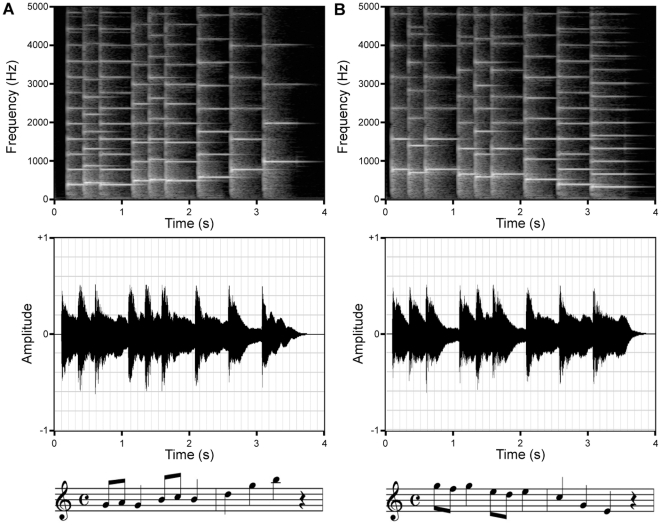
Spectrogram (top), envelope (middle) and score (bottom) of the Control Ascending Melody (A) and the Experimental Descending Melody (B).

Two melodies were composed for the study. They were played on a piano, synthesized with MIDI software, stored on a PC and normalized (Cool Edit Pro software). The melodies have been previously tested in our lab. They reliably elicited small HR decelerations in 37–38 weeks GA fetuses during quiet sleep at the sound pressure level that was used for the familiarization sessions (S.Bassereau, MS thesis, 1998; A.Ribeiro, MS thesis, 2000). Each melody lasted 3.6 s and had nine notes from the same two octave bands, C4 and C5. The Experimental Melody had a descending melodic contour, from G5 (784 Hz) to E4 (330 Hz) and the Control Melody had an ascending melodic contour, from G4 (392 Hz) to B5 (988 Hz). Although the magnitude of pitch change from one note to the next increased from the beginning to the end of each melody in opposite directions, the octave-change ratios of successive notes were identical, viz., 1/8, 1/8, 2/8, 1/8, 1/8, 2/8, 3/8 and 2/8. Constructing inverse melodic contours while keeping their change ratios the same allowed four of the nine notes to be identical but required the other five notes to differ slightly between the melodies. The total “on” time of the melodies was the same and the series of consecutive eighth notes and quarter notes were identical, giving the melodies the same tempo, rhythm, and thus, almost identical amplitude envelopes. In sum, the piano melodies had similar spectra, almost identical amplitude envelopes but precisely inverse melodic contours (Figure 2A and 2B). A “Stimulation Sequence” was defined as four repetitions of the 3.6 s melody each separated with 200 ms of silence, thus lasting 15 s.

#### Prenatal familiarization stimulus

A copy of the prenatal stimulus familiarization tape recording was given to the mother. It began with 5 min of recorded silence, followed by a 115 s sound period and ended with another 5 min of recorded silence. The sound period consisted of 6 Stimulation Sequences of the Experimental Melody, each separated by 5 s of silence. The SPL (re: 20 µPa) of the sequences gradually increased so as not to startle the mother. The first sequence was emitted at 60±2 dB Leq (62 dB peak level), the second sequence at 70±2 dB Leq (72 dB peak level) and the four following sequences at 80±2 dB Leq (82 dB peak level). The maximum SPL of the Experimental Melody, i.e., 80 dB Leq, is conservatively estimated to be 55–60 dB *in utero* (see Introduction). The order of events within a session, the 5 min silent periods before and after the stimulation period was contrived to isolate or differentiate the environmental characteristics surrounding the fetus's experience with the experimental melody, from environmental characteristics surrounding the day-to-day auditory experiences they have during the three weeks.

Each mother was given the same audio equipment to use at home, a Sony tape player and two small loudspeakers with an integrated amplifier (SP 681: 12.5 cm×9 cm×4 cm). The volume controls of the tape player and speakers were calibrated in the laboratory by measuring SPL with a sound level meter (ACLAN-SDH80) placed 5 cm from a mother's abdomen when the loudspeakers were placed at 50 cm from each side of her abdomen. The volume controls were locked and could not be changed by the mothers. The materials were retrieved at the end of the three weeks familiarization period.

#### Postnatal testing stimulus

During postnatal testing, the 15 s Stimulation Sequence of the Experimental and Control melodies was delivered at 60±2 dB Leq, 62 dB peak level, measured with the sound level meter placed where the infants' head would be inside the testing chamber (see procedure). Background noise inside the chamber was 45±2 dB SPL. Sound pressure level of the testing stimulus was checked before each test.

### Procedure

#### Prenatal familiarization sessions

The Experimental subjects were exposed to the Experimental Melody in the mother's home during the 35^th^, 36^th^, and 37^th^ weeks of gestation. Two familiarization sessions occurred each day with a minimum of 5 hours delay between sessions. Mothers were asked to be comfortably seated or lying in a silent room between the two loudspeakers, placed at about 50 cm from their abdomen, to relax and not to make any noise, speak or touch their abdomen. They were allowed to read silently. Each exposure session ran automatically for 12 minutes when the mother started the tape player. If the session was interrupted for any reason the mothers were told to stop the familiarization session and start it again at least 5 minutes later. After each session they filled out a log describing the conditions and time of the session, how they felt physically, how much they felt their fetus move and what they did during the hour before the session.

In principle, over 21 days of exposure a fetus would experience 1008 repetitions of the 3.6 s Experimental Melody (42 sessions×24 occurrences/session). If only the loudest Stimulation Sequences, at 80 dB SPL, are considered, i.e., the ones most likely to be heard by a fetus, there would be 672 repetitions of the Experimental Melody (42 sessions×16 occurrences/session). In fact, the mean number of exposure sessions that occurred in the final Experimental Group was 39.24, SD = 2.05 (range = 35–42 sessions), for a mean of 942 total presentations of the 3.6 s Experimental Melody, among them 628 presentations at 80 dB SPL. The mean retention interval, mean time between the last prenatal familiarization session and postnatal testing, was 44 days (6 weeks and 2 days), SD = 2.62, range = 41–50 days.

#### Postnatal testing

The mothers entered the laboratory that was quiet and dimly lighted, so as to not wake up sleeping infants and to facilitate the onset of sleep in infants who were awake. They were reminded of the details of the testing procedure and could ask questions. The transducer (7.3 cm in diameter) of a Doppler cardiotocograph (Hewlett-Packard M1351A), connected to a PC computer, was placed on the infant's chest and held in place by a stretched cloth belt. Room-temperature almond oil was used as a contact product for the transducer instead of regular gel whose chilling effect could startle or wake the infants. If the infant was awake, we relied on the mother's judgment about the best way to put her infant to sleep (all infants stayed in their mother's arms, some were breastfed or had a pacifier). When asleep, the infant was seated in a semi-reclined position in an infant chair that was placed inside a sound attenuating chamber (62 cm high×57 cm wide×64 cm long) with the infant's head facing the opening in the front (58 cm high×53 cm wide) to allow observation by the experimenters. A piece of cloth that retained the infant's or mother's odor, e.g., a sheet, blanket or a piece of clothing, was placed alongside the infant's head and neck. Inside the chamber two small loudspeakers like the ones used for the prenatal familiarization sessions were located on each side of the infant's head, 20 cm from each ear.

Testing started when two experimenters agreed that the infant had been in quiet sleep for 5 consecutive minutes, with a stable low variability HR (SD≤3 bpm), regular slow breathing movements, eyelids closed with no REM, no body movements and no muscle tone [95]. A low moment to moment variability baseline HR allows more reliable detection of low magnitude stimulus-elicited cardiac responses than do the highly variable HRs of active sleep or wakefulness. Then, one “Stimulation Sequence” of either the Experimental Melody or the Control Melody was delivered at 60 dB SPL.

Infant HR was recorded continuously during the 5 min before stimulation, during stimulation and the 5 min after stimulation, to see whether behavioral state changed. Two experimenters monitored the continuous analog HR tracing in beats per minute (bpm) that appeared on the PC screen, along with a display of its mean and standard deviation that was updated every 30 s, and on the paper printout of the cardiotocograph (paper speed = 2 cm/m). A proprietary software program (C. Kervella & R. Humbert) controlled stimulus presentations and sampled the analog cardiac signal at a rate of 10 times/second, digitized and stored each value online.

After the test session, all mothers answered a questionnaire regarding the infant's health and development and to verify that the daily acoustic characteristics of their home had nothing in particular that could potentially affect the infants' reactions to the test stimuli (e.g., regular unusually high noise levels in the home, or family members listening to or practicing the piano daily).

#### Data analysis

Three experienced observers (C.G-D., A-Y.J., S.B. or A.R.) examined each cardiac paper tracing to verify that the infant's HR pattern corresponded to a quiet sleep pattern 5 min before, during and 5 min after stimulation. The subject was retained only if the three observers agreed. The raw digital HR values were checked for missing or erroneous values, which were corrected by linear interpolation. Next, we checked whether stimulus-elicited cardiac responses occurred during the 15 s Stimulation Period. For each subject, the 150 (10/s) digital HR values of the 15 s Prestimulus Period (15 s preceding stimulus onset) were converted to z-scores**:** Our criterion for a response was that the subject's HR during the 15 s Stimulation Period increased by at least 3 SD (z≥+3) for a cardiac acceleration, or decreased by at least 3 SD (z≤−3) for a cardiac deceleration, from the Prestimulus Period, and remained at that level for at least two consecutive seconds. The 300 digital HR data were also analyzed with parametric (mixed ANOVAs) and non parametric (Chi-Square and Mann-Whitney U test) statistics with Statistica 8 software.

## References

[1] Pujol R, Laville-Rebillard M, Lenoir M, Rubel EW, Popper AN, Fay RR (1998). Development of sensory neural structures in the Mammalian cochlea.. Development of the auditory system.

[2] Morlet T, Collet L, Salle B, Morgon A (1993). Functional maturation of cochlear active mechanisms of the medial olivocochlear system in humans.. Acta Otolaryngol (Stock).

[3] Rotteveel JJ, de Graaf R, Stegeman DF, Colon EJ, Visco YM (1987). The maturation of the central auditory conduction in preterm infants until three months post term V: The auditory cortical response (ACR).. Hear Res.

[4] Eldredge L, Salamy A (1996). Functional auditory development in preterm and full term infants.. Early Hum Dev.

[5] Lecanuet J-P, Granier-Deferre C, Busnel M-C (1988). Fetal cardiac motor responses to octave–band noises as a function of central frequency, intensity and heart rate variability.. Early Human Dev.

[6] Lecanuet J-P, Granier-Deferre C, DeCasper AJ, Maugeais R, Andrieu AJ (1987). Perception et discrimination foetales de stimuli langagiers: mise en évidence à partir de la réactivité cardiaque, résultats préliminaires.. C R Acad Sci Paris Ser III.

[7] Lecanuet J-P, Granier-Deferre C, Busnel M-C (1989). Differential fetal auditory reactiveness as a function of stimulus characteristics and state.. Sem in Perinat.

[8] Lecanuet J-P, Granier-Deferre C, Jacquet A–Y, Busnel M-C (1992). Decelerative cardiac responsiveness to acoustical stimulation in the near term foetus.. Quart J of Exp Psychol.

[9] Lecanuet J-P, Granier-Deferre C, Jacquet A–Y, Capponi I, Ledru L (1993). Prenatal discrimination of a male and a female voice uttering the same sentence.. Early Dev Parenting.

[10] Lecanuet J-P, Granier-Deferre C, Jacquet A-Y, DeCasper AJ (2000). Fetal discrimination of low–pitched musical notes.. Dev Psychobiol.

[11] Shahidullah S, Hepper PG (1994). Frequency discrimination by the fetus.. Early Human Dev.

[12] Groome LJ, Mooney DM, Holland SB, Smith LA, Atterbury JL (1999). Behavioral state affects heart rate response to low-intensity sound in human fetuses.. Early Human Dev.

[13] Granier-Deferre C, Ribeiro A, Jacquet A-Y, Bassereau S (2010). Near–term fetuses process temporal features of speech.. Dev Sci.

[14] Lecanuet J-P, Jacquet A-Y (2002).

[15] Lecanuet J-P, Granier-Deferre C, Busnel M-C, Lecanuet J-P, Fifer WP, Krasnegor NA, Smotherman WP (1995). Human fetal auditory perception.. Fetal development: a psychobiological perspective.

[16] Schleussner E, Schneider U (2004). Developmental changes of auditory-evoked fields in fetuses.. Exp Neurol.

[17] Holst M, Eswaran H, Lowery C, Murphy P, Norton J (2005). Development of auditory evoked fields in human fetuses newborns: a longitudinal MEG study.. Clin Neurophysiol.

[18] Huotilainen M, Kujala A, Hotakainen M, Parkkonen L, Taulu S (2005). Short–term memory functions of the human fetus recorded with magnetoencephalography.. Neuroreport.

[19] Draganova R, Eswaran H, Murphy P, Lowery C, Preissl H (2007). Serial magnetoencephalographic study of fetal newborn auditory discriminative evoked responses.. Early Hum Dev.

[20] Moore RJ, Vadeyar S, Fulford J, Tyler DJ, Gribben C (2001). Antenatal determination of fetal brain activity in response to an acoustic stimulus using functional magnetic resonance imaging.. HBM.

[21] Fulford J, Vadeyar SH, Dodampahala SH, Ong S, Moore RJ (2004). Fetal brain activity and hemodynamic response to a vibroacoustic stimulus.. HBM.

[22] Jardri R, Pins D, Houfflin–Debarge V, Chaffiotte C, Rocourt N (2008). Fetal cortical activation to sound at 33 weeks of gestation: a functional MRI study.. NeuroImage.

[23] Armitage SE, Baldwin BA, Vince MA (1980). The fetal sound environment of sheep.. Science.

[24] Peters AJ, Abrams RM, Gerhardt KJ, Griffiths SK (1993). Transmission of airborne sounds from 50–20,000 Hz into the abdomen of sheep.. J Low Freq Noise Vib.

[25] Querleu D, Renard X, Versyp F, Paris-Delrue L, Vervoot P (1988). La transmission intra-amniotique des voix humaines.. Rev Gynecol Obstet.

[26] Querleu D, Renard X, Boutteville C, Crépin G (1989). Hearing by the human fetus?. Semin Perinatol.

[27] Benzaquen S, Gagnon R, Hunse C, Foreman J (1990). The intrauterine sound environment of the human fetus during labor.. Am J Obstet Gynecol.

[28] Richards DS, Frentzen B, Gerhardt KJ, McCann ME, Abrams RM (1992). Sound levels in the human uterus.. Obstet Gynecol.

[29] Griffiths SJ, Brown WS, Gerhardt KJ, Abrams RM, Morris RJ (1994). The perception of speech sounds recorded within the uterus of a pregnant sheep.. JASA.

[30] Abrams RM, Griffiths SK, Huang X, Sain J, Langford G (1998). Fetal music perception: The role of sound transmission.. Music Percept.

[31] Rovee-Collier C, Cueva SK (2009). Multiple memory systems are unnecessary to account for infant memory development: an ecological model.. Dev Psy.

[32] Granier-Deferre C, Lecanuet J-P, Cohen H, Busnel M-C (1985). Feasibility of prenatal hearing test.. Acta–Otolaryngol (Stockh).

[33] Lecanuet J-P, Granier-Deferre C, Cohen H, Le Houezec R, Busnel M-C (1986). Fetal responses to acoustic stimulation depend on HR variability pattern, stimulus intensity and repetition. Early Human Dev.

[34] Hepper PG (1996). Fetal memory: Does it exist? What does it do?. Acta Paediatr.

[35] Leader LR, Baillie P, Martin B, Vermeulen E (1982). The assessment and significance of habituation to a repeated stimulus by the human fetus.. Early Human Dev.

[36] Madison LS, Adubato SA, Madison JK, Nelson RM, Anderson JC (1986). Fetal response decrement: True habituation?. J Dev Behav Pediat.

[37] Kisilevsky BS, Muir DW, Low JA (1992). Maturation of human fetal responses to vibroacoustic stimulation.. Child Dev.

[38] Visser GH, Mulder EJ (1993). The effect of vibro-acoustic stimulation on fetal behavioral state organization.. Am J Ind Med.

[39] Smith CV (1995). Vibroacoustic stimulation.. Clin Obstet Gynecol.

[40] Sandman CA, Wadhwa PD, Hetrick WP, Porto M, Peeke HVS (1997). Human fetal heart rate dishabituation between 30–32 weeks gestation.. Child Dev.

[41] Sandman CA, Glynn L, Wadhwa PD, Chicz-DeMet A, Porto M (2003). Maternal HPA deregulation during the third trimester influences human fetal responses.. Dev Neurosci.

[42] van Heteren CF, Boekkooi P, Jongsma HW, Nijhuis JG (2000). Fetal learning memory Lancet.

[43] Dirix CE, Nijhuis JG, Jongsma HW, Hornstra G (2009). Aspects of fetal learning memory.. Child Dev.

[44] Gonzalez-Gonzalez NL, Suarez MN, Perez-Pinero B, Armas H, Domenech E (2006). Persistence of fetal memory into neonatal life.. Acta Obstet Gynecol Scand.

[45] Graham EM, Peters AJ, Abrams RM, Gerhardt KJ, Burchfield DJ (1991). Intraabdominal sound levels during vibroacoustic stimulation.. Am J Obstet Gynecol.

[46] Abrams RM, Peters AJ, Gerhardt KJ (1997). Effect of abdominal vibroacoustic stimulation on sound acceleration levels at the head of the fetal sheep.. Obstet Gynecol.

[47] Ando Y, Hattori H (1977). Effects of noise on sleep of babies.. JASA.

[48] Spelt DK (1948). The conditioning of the fetus in utero.. J Exp Psychol.

[49] Kawai N, Morokuma S, Tomonaga M, Horimoto N, Tanaka M (2004). Associative learning memory in a chimpanzee fetus: learning long–lasting memory before birth.. Dev Psychobio.

[50] James DK, Spencer CJ, Stepsis BW (2002). Fetal learning: a prospective randomized controlled study.. Ultrasound Obstet Gynecol.

[51] DeCasper AJ, Lecanuet J-P, Maugeais R, Granier-Deferre C, Busnel M-C (1994). Fetal reactions to recurrent maternal speech.. Inf Behav Dev.

[52] Krueger C, Holditch-Davis D, Quint S, DeCasper AJ (2004). Recurring auditory experience in the 28– to 34–week old fetus.. Inf Behav Dev.

[53] Kisilevsky BS, Hains SM, Lee K, Xie X, Huang H (2003). Effects of experience on fetal voice recognition.. Psychol Sci.

[54] Smith LS, Dmochowski PA, Muir DW, Kisilevsky BS (2007). Estimated cardiac vagal tone predicts fetal responses to mother's stranger's voices.. Dev Psychobio.

[55] DeCasper AJ, Spence MJ, Weiss MJ, Zelazo PR (1991). Auditorially mediated behavior during the perinatal period: A cognitive view.. Infant Attention.

[56] DeCasper AJ, Fifer WP (1980). Of human bonding: newborns prefer their mother's voice.. Science.

[57] Spence MJ, DeCasper AJ (1987). Prenatal experience with low frequency maternal voice sounds influences neonatal perception of maternal voice samples.. Infant Behav Dev.

[58] Spence MJ, Freeman MS (1996). Newborn infants prefer the maternal low-pass filtered voice, but not the maternal whispered voice.. Inf Behav Dev.

[59] Fifer WP, Moon CM, Lecanuet J-P, Fifer WP, Krasnegor NA, Smotherman WP (1995). Effects of fetal experience with sound.. Fetal development: a psychobiological perspective.

[60] DeCasper AJ, Spence MJ (1986). Prenatal maternal speech influences newborn's perception of speech sounds.. Infant Behav Dev.

[61] Cooper RP, Aslin RN (1989). The language environment of the young infant: Implications for early perceptual development.. Canad J Psychol.

[62] Moon CM, Cooper R, Fifer WP (1993). Two–Days–olds prefer their native language Infant.. Behav Dev.

[63] DeCasper AJ, Prescott P (2009). Lateralized processes constrain auditory reinforcement in human newborns.. Hear Res.

[64] Byers-Heinlein K, Burns TC, Werker JF (2010). The roots of bilingualism in newborns.. Psychol Sci.

[65] Mastropieri D, Turkewitz G (1999). Prenatal experience and neonatal responsiveness to vocal expressions of emotion.. Dev Psychobio.

[66] Hepper PG (1988). Fetal “soap”addiction.. Lancet.

[67] Trehub ST (2001). Musical predispositions in infancy.. Ann NY Acad Sci.

[68] Fernald A, Kuhl P (1997). Acoustic determinants of infant preference for motherese.. Inf Behav Dev.

[69] Porges SW (1974). Heart rate indices of newborn attentional responsiveness.. Merrill-Palmer Quarterly.

[70] Graham FK, Campbell BA, Hayne H, Richardson R (1992). Attention: The heart beat, the blink, the brain.. Attention: information processing in infants adults; perspective from human and animal research.

[71] Guyomarc'h J-C (1974). L'empreinte auditive prénatale.. Rev Comp Anim.

[72] Gottlieb G (1982). Development of species identification in ducklings: IX The necessity of experiencing normal variations in embryonic auditory stimulation.. Dev Psychobio.

[73] Granier-Deferre C, Lecanuet J-P (1987). Influence de stimulations auditives précoces sur la maturation anatomique et fonctionnelle du système auditif.. Prog Neonatol.

[74] Lickliter R, Stoumbos J (1992). Modification of prenatal auditory experience alters postnatal auditory preferences of bobwhite quail chicks.. Quart J Exp Psychol.

[75] Dmitrieva LP, Gottlieb G (1994). Influence of auditory experience on the development of brain stem auditory-evoked potentials in mallard duck embryos hatchlings.. Behav Neural Biol.

[76] Vince MA (1979). Postnatal effects of prenatal sound stimulation in the guinea pig.. Anim Behav.

[77] Keuroghlian AS, Knudsen EI (2007). Adaptive auditory plasticity in developing adult animals.. Prog Neurobiol.

[78] Sanes DH, Bao S (2009). Tuning up the developing auditory CNS.. Curr Opin Neurobiol.

[79] de Villers-Sidani E, Chang EF, Bao S, Merzenich MM (2007). Critical period window for spectral tuning defined in the primary auditory cortex (A1) in the rat.. J Neurosci.

[80] Xu J, Yu L, Cai R, Zhang J, Sun X (2009). Early auditory enrichment with music enhances auditory discrimination learning and alters NR2B protein expression in rat auditory cortex.. Behav Brain Res.

[81] Insanally MN, Kover H, Kim H, Bao S (2009). Feature-dependent sensitive periods in the development of complex sound representation.. J Neurosci.

[82] Weinberger NM (1998). Physiological memory in primary auditory cortex: characteristics mechanisms.. Neurobiol Learn Mem.

[83] Rayson P, Leech G, Hodges M (1997). Social differentiation in the use of English vocabulary: some analyses of the conversational component of the British National Corpus.. Int J Corpus Ling.

[84] Mehl MR, Vazire S, Ramírez-Esparza N, Slatcher RB, Pennebaker JW (2007). Are women really more talkative than men?. Science.

[85] Seebach BS, Intrator N, Lieberman P, Cooper LN (1994). A model of prenatal acquisition of speech parameters.. Proc Natl Acad Sci USA.

[86] Teinonen T, Fellman V, Näätänen R, Alku P, Huotilainen M (2009). Statistical language learning in neonates revealed by event-related brain potentials.. BMC Neurosci.

[87] Lecanuet J-P, Granier-Deferre C, de Boysson–Bardies B, de Schoenen S, Jusczyck P, McNeilag P, Morton EJ (1993). Speech stimuli in the fetal environment.. Developmental neurocognition: speech face processing in the first year of life.

[88] Moore JK (2002). Maturation of human auditory cortex: Implications for speech perception.. Ann Otol Rhinol Laryngol.

[89] Nazzi T, Bertoncini J, Mehler J (1998). Language discrimination by newborns: towards an understanding of the role of rhythm.. J Exp Psychol: Hum Percep Perf.

[90] Mampe B, Friederici AD, Christophe A, Wermke K (2009). Newborns' cry melody is shaped by their native language.. Current Biol.

[91] Smotherman WP (1982). In utero chemosensory experience alters taste preference and corticosteroid responsiveness.. Behav Neural Biol.

[92] Leon M, Coopersmith R, Lee S, Sullivan RM, Wilson DA, Krasnegor N, Blass E, Hofer M, Smotherman W (1987). Neural and behavioral plasticity induced by early olfactory learning.. Perinatal development: a psychobiological perspective.

[93] Schaal B, Marlier l, Soussignan R (2000). Human fetuses learn odors from their pregnant mother's diet.. Chem Senses.

[94] Marlier L, Schaal B (2005). Human newborns prefer human milk: conspecific milk odor is attractive without postnatal exposure.. Child Dev.

[95] Prechtl HF (1974). The behavioral states of the newborn infant (a review).. Brain Res.

